# Social origin and the intention to enrol in higher education: personality traits as a mechanism of reproduction or mobility?

**DOI:** 10.3389/fsoc.2025.1652429

**Published:** 2025-08-22

**Authors:** David Nika, Michael Grüttner, Sandra Buchholz

**Affiliations:** German Centre for Higher Education Research and Science Studies, Hannover, Germany

**Keywords:** educational attainment, higher education enrolment, personality traits, *resource substitution*, structural equation modelling

## Abstract

A vast amount of research has shown that social inequality in educational attainment is a persistent phenomenon. Sociological research explains unequal educational decisions via primary and secondary effects of social origin, respectively unequal school performance and patterns of educational decision-making. So far, educational sociology has largely ignored the role of personality traits for educational decision-making. Therefore, we extend the sociological perspective on primary and secondary effects of social origin to include personality traits as non-cognitive resources. Three plausible mechanisms could be at work: (1) For students from low social origins, favourable personality traits could compensate for the lack of other important resources and be more important for their study intention (*resource substitution*). (2) Although students from low social origins benefit most from personal traits, they lack precisely these personal resources (*structural amplification*). (3) Students from high social origins have more favourable personality traits and can also profit more from them (*resource multiplication*). Using data from the DZHW Panel Study of School Leavers with a Higher Education Entrance Qualification, we estimate a structural equation model (SEM) to examine the direct, indirect, and total effects of personality traits on the intention to enrol in higher education. Results are twofold: First, personality traits are significant determinants of primary and secondary effects of social origin. Second and most importantly, openness proves to be a key resource: while students from less advantaged social origin generally display lower levels of openness, this group benefits most from this trait in forming their intention to pursue higher education—a pattern consistent with structural amplification. These results highlight the dual role of personality traits in both enabling individual upward mobility and contributing to the persistence of social inequality. The study underscores the importance of considering non-cognitive resources in explanations of educational inequality and points to potential interventions aimed at fostering openness.

## 1 Introduction

Social inequality in educational attainment is a persistent phenomenon in modern societies (Boliver, 2011; Breen et al., 2009; Shavit and Blossfeld, 1993), which also hold true for children’s chances to participate in higher education in Germany (Thompson, 2009, 2017). Although Germany has experienced a substantial increase in university enrolment rates since the start of the new millennium, with nearly half of young adults now entering higher education, the effects of social origin at the transition to higher education has barely changed (Quast et al., 2023; Schindler and Lörz, 2012).

There is a longstanding tradition in sociological research to explain social origin effects in education as a result of both, differences in children’s educational performance and individuals’ educational decisions (Boudon, 1974; Breen and Goldthorpe, 1997). The central argument is that children from advantaged backgrounds profit from more supportive learning environments due to their parents’ higher cultural and socioeconomic resources (Becker, 2019), which in turn leads to better school performance (so-called primary effect of social origin). However, even when children perform equally well at school, their social origin has been found to make a difference. This has been labelled the secondary effect of social origin, arising from social disparities in educational decision-making. Those from lower social background perceive higher costs, lower returns and lower probabilities of success of investing in education, compared to those from more privileged backgrounds (Becker, 2022). For the transition to higher education, it has been shown that it is in particular disparities in educational decision-making explaining inequalities in college enrolment (Quast et al., 2023).

Related disciplines of educational economics and psychology have already argued for the importance of personality traits for socially unequal educational trajectories (Coenen et al., 2021; Cunha and Heckman, 2007, 2008; Heckman and Mosso, 2014) and have shown remarkable effects on educational performance (Edwards et al., 2022). However, sociological research has barely provided any explanations as to which personality traits might help children from disadvantaged backgrounds overcome barriers to university enrolment and decide for higher education. This is remarkable, for with habitus (Bourdieu, 1984, 1986), the sociological tradition provides a concept that can serve as an argument for thinking social structure and personality together (Kaiser and Diewald, 2014b; Schmitz and Barth, 2018). Although the concept of habitus is central for Bourdieu’s sociological theory and sociological research has already established meaningful relations between primary and secondary effects and other concepts of Bourdieu, such as cultural capital (Nash, 2003; Stocké, 2013; Van De Werfhorst and Hofstede, 2007), links to the concept of personality have hardly been established (Schmitz and Barth, 2018). For educational performance (primary effect of social origin), there are some studies that use the habitus concept to argue for the importance of personality traits (Kaiser and Diewald, 2014a; Kaiser and Schneickert, 2016), but corresponding investigations for educational decision-making (secondary effect of social origin) are still pending. Studies on late educational transitions, which are more strongly influenced by the personal decisions of young adults than by the decisions of parents and teachers, seem particularly suitable for this purpose. Therefore, our empirical study aims to gain a deeper understanding of the role of personality traits in explaining social inequalities in university enrolment.

We aim to connect personality traits to the approach of primary and secondary effects of social origin using the example of socially unequal transitions from German high schools into higher education. We investigate the research question of how personality traits impact the intention to enrol in higher education. Thereby, we seek to avoid a deficit perspective on students from less advantaged social origins and to investigate potential resources that may facilitate “success against the odds” (Solga et al., 2013). Therefore, we rely on a resource perspective (Ross and Mirowsky, 2011; Stienstra et al., 2021) and include personality traits as personal resources that students build on when investing in educational achievement and decision-making. According to Ross and Mirowsky (2011), three considerations are of central importance: the *resource substitution* hypothesis would suggest that students from less advantaged social origins could compensate for social disadvantages through favourable endowment with personality traits. Moreover, personality traits may play a greater role in the educational attainment of socially disadvantaged student groups than of those groups with more advantageous backgrounds. In contrast, *resource multiplication* would mean that students from more advantaged social origins would profit more from their personality traits than those from less advantageous social origins. With regard to *structural amplification*, students from less advantageous social origins would have lower levels of endowment with favourable personality traits, in combination with stronger effects of personality on educational decisions. On this basis, we ask to what extent personality traits are related to social inequality in higher education enrolment in Germany. Depending on whether the former hypothesis or one of the latter two is valid, personality traits serve as a mechanism of reproduction or mobility.

Therefore, we model primary and secondary effects of social origin and investigate the direct, indirect, and total effects of personality traits on the intention to enrol in higher education after obtaining a HEEQ. We use data from the DZHW 2018 panel study of school leavers 6 months before and 6 months after high school graduation (Woisch et al., 2019). We employ structural equation modelling to analyse theoretically meaningful paths between personality traits (McCrae and Costa, 1996), educational achievement, educational choice variables, and the intention to enrol in higher education.

## 2 Literature and theory

### 2.1 The role of primary and secondary effects of social origin in education

Sociology has a long tradition of studying inequalities in education, and it is well documented that social origin effects have largely persisted in modern societies despite the enormous educational expansion of the past century (e.g., Becker, 2009; Jackson, 2013; Maaz, 2006; Müller and Pollak, 2004; Schindler and Lörz, 2012; Schindler and Reimer, 2010; Watermann et al., 2014). Children’s educational opportunities are still closely linked to their family of origin.

Probably the most frequently applied and prominent theoretical model builds on the pioneering work of Boudon (1974) who introduced the differentiation between primary and secondary effects of social origin. His central argument is that social origin impacts children’s educational opportunities and attainment via two different “channels”. First, social origin matters in education, because the family of origin has a direct effect on the child’s development of abilities and academic performance, for example by providing very different learning environments (so-called primary effect of social origin). Secondly, even when children’s abilities and academic performance are the same, social origin still makes a difference because it shapes individuals’ educational decision-making (so-called secondary effect of social origin). These differences in educational decisions – irrespective of children’s academic performance and abilities – are explained by the fact that the costs, returns and probabilities of success of educational options are perceived quite differently by different groups of social origin (Breen and Goldthorpe, 1997; Erikson and Jonsson, 1996; Esser, 1999).

There is sound empirical evidence that the model of primary and secondary effects is a powerful tool for explaining how educational inequality is transferred from parents to children (Becker, 2009; Jackson, 2013; Maaz, 2006; Müller and Pollak, 2004; Schindler and Lörz, 2012; Schindler and Reimer, 2010; Watermann et al., 2014). It has been shown, for a wide range of transition points in individual educational careers across a wide variety of countries, that driving factors for the intergenerational reproduction of inequality in education include not only origin-related disparities in children’s academic performance but also origin-specific differences in educational decision-making (Lörz, 2008; Schindler and Lörz, 2012; Schindler and Reimer, 2010; Watermann et al., 2014). Despite this evidence, we should, as Jackson (2013, p. 19) has argued, “certainly acknowledge that primary and secondary effects have complex causes.” In a recently published decomposition analysis, for example, Quast et al. (2023) have shown that origin-related disparities in the transition to higher education in Germany could be fully explained if models consider children’s school grades, perceived costs, returns and probabilities of success of higher education enrolment as well as parents’ and friends’ educational preferences. Quast and colleagues thereby combined the classical Boudon model with the Wisconsin model (Sewell et al., 1969; Zimmermann, 2019). The decomposition analysis additionally shows that although the primary effect of social origin is significant, it appears to play only a minor role in explaining inequalities in the transition to higher education. “Only” 20 per cent of the differences in college enrolment could be traced back to differences in children’s school grades. Aspects of the secondary effect – that is the perceived costs, perceived probability of success and returns of higher education, and the motive of intergenerational status maintenance – played a far greater role in explaining origin-related disparities in higher education enrolment after leaving school. In the decomposition analysis, these factors were able to explain more than 60 per cent of the effect of social origin. If we seek to better understand social inequalities in the transition that follows high school, it is therefore worth focusing on the secondary effect. Instead of combining different sociological models to investigate the “complex causes” (Jackson, 2013), as Zimmermann (2019) does, we focus on additional variables that are rarely considered in sociology of education so far, namely personality traits.

### 2.2 But what about individual personality traits?

While sociological research has put much emphasis on the role of (cognitive) abilities and what “rationalises” individuals’ educational decision-making, other disciplines, in particular microeconomics and psychology, have highlighted the importance of individuals’ non-cognitive resources (Cunha et al., 2006, 2010; Cunha and Heckman, 2008; Thiel and Thomsen, 2013) such as personality traits. One of the most established concepts of measuring personality traits as patterns of behavioural dispositions is the Big Five personality traits (McCrae and Costa, 1996, 1999), which can be differentiated as follows: individual (1) *openness* to experience, (2) *conscientiousness*, (3) *extraversion*, (4) *agreeableness* as well as (5) *emotional stability*. *Openness* covers aspects of personality such as willingness or appreciation of fantasies, new ideas and feelings, while *conscientiousness* covers aspects such as competence, orderliness, sense of duty or self-discipline. Aspects like sociability, drive, and enthusiasm are covered by *extraversion*. *Agreeableness* covers characteristics like modesty, cooperativeness, and altruism, while *emotional stability* covers aspects like lower levels anxiousness, insecurity, or irritability. While educational economics and psychology have already shown the strong influence of personality traits on educational performance (Edwards et al., 2022; Komarraju et al., 2009) we know little about the extent to which personality traits contribute to socially differentiated educational decisions via determinants of the secondary effect.

While the traits of conscientiousness, openness and emotional stability are usually positively associated with individuals’ educational attainment and outcomes (Berkes and Peter, 2019; Fuertes et al., 2020; Kajonius and Carlander, 2017; Komarraju et al., 2009; Palczyńska and Świst, 2018; Peter and Storck, 2015; Polemis, 2021; Rammstedt et al., 2017; Staff et al., 2017), results for extraversion and agreeableness are rather mixed. While some studies have shown no effects on the intention to study in higher education or on overall educational attainment (Berkes and Peter, 2019; Peter and Storck, 2015), others have shown positive effects on these outcomes (Fuertes et al., 2020; Kajonius and Carlander, 2017; Komarraju et al., 2009; Polemis, 2021) and yet others have shown negative effects of these traits (Palczyńska and Świst, 2018; Rammstedt et al., 2017).

Though there is clear evidence that individuals’ personality traits matter in education, sociological research has largely neglected their potential role in the intergenerational reproduction of inequality in education. This is striking, as there is sociological literature that shows class-specific values and child-rearing practises (Kohn, 1963; Kohn and Schooler, 1969; Lareau, 2002; Weininger and Lareau, 2009) that could instil psychological and behavioural patterns that are relevant to educational and economic attainment (Conger et al., 2021; Shanahan et al., 2014). Moreover, with the sociological concept of habitus Bourdieu (1984, 1995) has offered a bridge between social structure and individual patterns of behaviour. Bourdieu described a person’s habitus as a generative structure of patterns of perception, thought and action. He assumes that these habitual patterns are essentially shaped by the family of origin and the social class milieu and play a primary role for educational and social reproduction. As Schmitz and colleagues argue, both the concept of personality and habitus refer to a particular set of dispositions related to perception, reasoning and behaviour (Schmitz and Barth, 2018; Schmitz and Bayer, 2017). Schmitz and Barth (2018) rightly point out that Bourdieu never developed his own concept of personality traits in the sense of the Big Five, but nevertheless speaks of a more pronounced trait of openness in higher social classes and certain aspects of agreeableness and conscientiousness in lower social classes (Bourdieu, 1984). Although the psychological evidence on differences in personality traits depending on social origin is not uniform, numerous studies support observations comparable to Bourdieu’s (Ayoub et al., 2018; Conger et al., 2021; Flensborg-Madsen and Mortensen, 2014; Furnham and Cheng, 2014; Jonassaint et al., 2011; Kaiser, 2017; Kaiser and Diewald, 2014a,b; Sutin et al., 2017). According to this evidence, children from higher social origins tend to have more favourable endowments of openness, conscientiousness and emotional stability. For conscientiousness Conger et al. (2021) could observe transmissions across three generations. Kaiser and colleagues (Kaiser, 2017; Kaiser and Diewald, 2014b; Kaiser and Schneickert, 2016) showed how openness and certain favourable aspects of conscientiousness partly mediate the effects of social origin on school grades. This is not surprising since empirical findings demonstrate that individuals’ personality traits affect their academic performance. Literature reported strong positive effects of conscientiousness, moderate effects of emotional stability and openness for the average grades in school and higher education, while almost no effects for agreeableness and extraversion (Andersen et al., 2020; Bergold and Steinmayr, 2018; Brandt et al., 2020; Corazzini et al., 2021; Edwards et al., 2022; Kappe and Van Der Flier, 2012; Komarraju et al., 2009; Komarraju et al., 2011; Lechner et al., 2017; Libbrecht et al., 2014; Noftle and Robins, 2007; Poropat, 2009; Spengler et al., 2016; Trapmann et al., 2007). Moreover, personality might not only be relevant for academic performance but also for educational decision making. To date, however, this question has hardly been investigated. Our literature review revealed only two studies that analyse the relationship between personality traits and variables that measure mechanisms of the secondary effect of social origin. Coenen et al. (2021) found a significant effect of personality on the expectations of educational success, and Ham et al. (2009) were able to show relationships between personality and desired occupations. This means that sociology has yet to systematically analyse the role of personality traits in the secondary effect of social origin for educational decisions.

Although individuals’ personality traits might be relevant mediators of social origin effects and important background factors for their academic performance and educational decision-making, they might be of even greater importance for groups with less advantaged social origins in order for them to enter education against the odds. We are thus adopting a similar perspective to that already applied by the second generation of Bourdieuian cultural capital research around Di Maggio and colleagues (Davies and Rizk, 2018). But with Mirowsky and Ross (2003) and Ross and Mirowsky (2011), we can identify three potential scenarios. First, with the *resource substitution* hypothesis (Mirowsky and Ross, 2003; Ross and Mirowsky, 2011) we can argue that personality traits could be potential resources that individuals can use to overcome social disadvantages during educational transitions. According to the *resource substitution* hypothesis, some resources can become more relevant for individuals if they lack certain other important resources. As we argued, based on the literature, individuals from socially disadvantaged backgrounds do have lesser cultural and economic resources which lead to a lower probability of their enrolling in higher education. Therefore, favourable endowments of certain personality traits could become more relevant for this group, meaning that these traits could have stronger positive effects on study intention- and decisions than for those with socially more advantaged backgrounds. Peter and Storck (2015) show some evidence for that assumption, but without relating their analysis to mechanisms of secondary effects of social origin. However, second, as the research has shown, individuals from socially disadvantaged groups tend to have a less favourable endowment of these personality traits. So, even if the effects of these traits are stronger for this group, they might benefit less often from it (*structural amplification*, Ross and Mirowsky, 2011). According to Ross and Mirowsky “structural amplification is a special case of *resource substitution* occurring when social conditions decrease the likelihood of attaining personal resources that otherwise would moderate the conditions undesirable consequences” (Ross and Mirowsky, 2011, p. 592). Results for this could be shown for emotional stability (Shanahan et al., 2014). Further, a third scenario is also possible, and can be described as *resource multiplication* (Ross and Mirowsky, 2011) or the Matthew effect (Merton, 1968). This means that individuals from socially advantaged origins could benefit even more from favourable endowments of certain personality traits, increasing the probability of enrolling in higher education even more in comparison to those from less socially advantaged groups. Again with reference to Bourdieu (1986) and Davies and Rizk (2018), the question is to what extent personality traits such as openness to experience serve as a mechanism of reproduction or mobility.

As we have highlighted in section 2.1 sociological research is able to explain most of the effects of social origin in higher education enrolment. However, not much emphasis has yet been placed on finding out how some children from less advantaged social origins are still able to enter university against the odds (Solga et al., 2013). Therefore, our research question is: *how are children from less advantaged social origins able to overcome social inequalities in education?* As we pointed out in this chapter, personality traits could be amongst the resources that help those individuals overcome inequalities. Therefore, to address our overall research question, we are looking to answer two corresponding questions: (1) Are performance, expected costs, returns and expectations of attending university influenced by personality traits? (2) Are personality traits more important for the study intention of students from less advantaged social origins than for those from more advantaged social origins (*resource substitution*)?

## 3 Data and methods

### 3.1 Data and dependent variable

We are using data from the DZHW 2018 Panel Study of School Leavers to perform our analyses. The sample for this data consists of German high school students who received their higher education entrance qualification (“Abitur”) in the school year 2017/2018. Data were collected via a disproportionate, random cluster sample. Currently, data are available for two waves of the survey and were collected about 6 months before and 6 months after graduation from high school. The surveys include questions on a variety of themes, such as attitudes, personality traits, social background, as well as educational and career decisions and intentions.

The dependent variable is the graduates’ intention to enrol in a higher education institution (study intention), which was measured in the first wave of the survey. Due to data availability, we are not using the actual study decision, because it was measured only 6 months after graduation. In this short period, many students in Germany do not finally decide in favour or against enrolling in higher education (Authoring Group Educational Reporting, 2020). Further, research shows a tight correlation between intention to enrol and actually enrolling in higher education (Quast et al., 2014). Therefore, it is reasonable to use the graduates’ intention as the dependent variable. This dichotomous variable is differentiated between 0 “no study intention” and 1 “study intention.” In our sample, 21.96% of the respondents report no study intention, while 78.04% report intending to enrol in higher education.^1^

Germany has a highly stratified educational system and different potential pathways towards or away from higher education (Allmendinger, 1989), and therefore differences in educational decisions. Thus, in this paper we are focusing only on “traditional” high school graduates. Therefore, we are excluding cases, which received their higher education entrance qualification in types of schools other than general high schools (“allgemeinbildende Schule”). Hence, respondents who did vocational training before or during high school, are also excluded. Further, we exclude cases with missing values on the variables included in our analyses. Thus, we have an analytic sample of 5,877 high school graduates.

### 3.2 Independent and control variables

The international socio-economic index of occupational status (ISEI-08) of each parent of the respondents is used as a measurement for the social origin of the high school graduates (Ganzeboom et al., 1992; Ganzeboom, 2010). This classification orders the occupations into a social hierarchy to specify to what extent it is possible to convert education into income with different professions. The scores ranges from 11.01 (farming for own consumption) up to 88.97 (judge). For the analysis we are comparing the ISEI-scores of each parent and using the higher score (HISEI) for our analyses. Afterwards the HISEI is categorised into three groups: low (25% or lower), middle (up to 75%) and high SES (higher than 75%), whereas a low SES indicates a disadvantaged social background.

The “Big Five” personality traits (McCrae and Costa, 1996, 1999) are used to analyse the effects of personality traits of the high school graduates on their study intention. Five dimensions are covered by the Big Five, including openness, conscientiousness, extraversion, agreeableness and emotional stability, which were measured with a inventory of 15 items (Schupp and Gerlitz, 2008) in the 2018 Panel Study of School Leavers. Each of the personality traits contain factor values, with a mean of 0 and a standard deviation of 1.

Primary effects are operationalised through the average grade in school 6 months prior to graduation (“Abiturnote”).^2^ We are inverting the values so that a higher value equals a better grade and vice versa. We use a series of different measures to operationalise the secondary effect of social origin. The expectation of success is measuring the respondents’ evaluation of whether they can successfully graduate from a higher education institution. Further, we measure the expected costs of studying with two variables. First, we operationalise the monetary costs of studying, containing aspects of the role of costs for the study intention, how difficult it would be for the respondents and their families to bear different costs during studying, as well as opportunity costs in terms of income loss through enrolling in higher education (*α* = 0.66). Second, we measure the social costs via the meaning of living near one’s hometown, and on whether parents, relatives, or friends are living near the place of study and whether these aspects are of importance for the choice of place of study (α = 0.80).

Further, we operationalise the expected benefits^3^ of studying through different variables: We measure different outlooks for the respondents according to whether they pursue higher education or vocational training after graduating from high school. These outlooks are: achieving a well-paying job, achieving a prestigious job, achieving an interesting job, and not becoming unemployed. Positive values indicate that the respondents perceive these outcomes as more achievable through higher education, while negative values indicate that these outcomes are more achievable via vocational training.

As control variables, we consider gender (male vs. female) and migration background (no vs. yes) in our analyses, as these variables have effects on the intention towards and decision regarding higher education (Kristen et al., 2008; Lörz et al., 2011; Mentges, 2019). All continuous variables are z-standardised and therefore have a mean of 0 and a standard deviation of 1.^4^

### 3.3 Analytic methods and strategy

Since we are assuming direct effects of the personality traits on study intention, as well as mediation through different intervening variables, so-called indirect effects (Hayes, 2009), we estimate a structural equation model (SEM) to test our assumptions. Contrary to the conventional use of logistic regressions to measure educational decisions, we can use SEM to measure the direct, indirect, and total effects of personality traits of the graduates’ study intention all at once (Hayes, 2009). In addition, we can measure covariances between certain variables (e.g., between cost or return variables). Further, SEM allows us to examine how well our model fits the observed data. Thus, this method allows us to explore the complex relationship between our variables. Since we are interested in group differences of the effect of personality on study intention, we estimate group models for the low, middle and high SES groups. With this we can explore whether certain social groups benefit more from the effect of personality traits than others.

Since we have a dichotomous outcome variable, we use a robust weighted least-squares estimator (WLSMV), since it is not dependent on the normality assumption (Kline, 2011). To perform our analyses, we use the statistical software R (R Core Team, 2022). The Package “lavaan” (Rossel, 2012) allows us to perform SEM in a similar manner as specialised software, such as Mplus. Results are reported as standardised coefficients.

**Figure 1:**
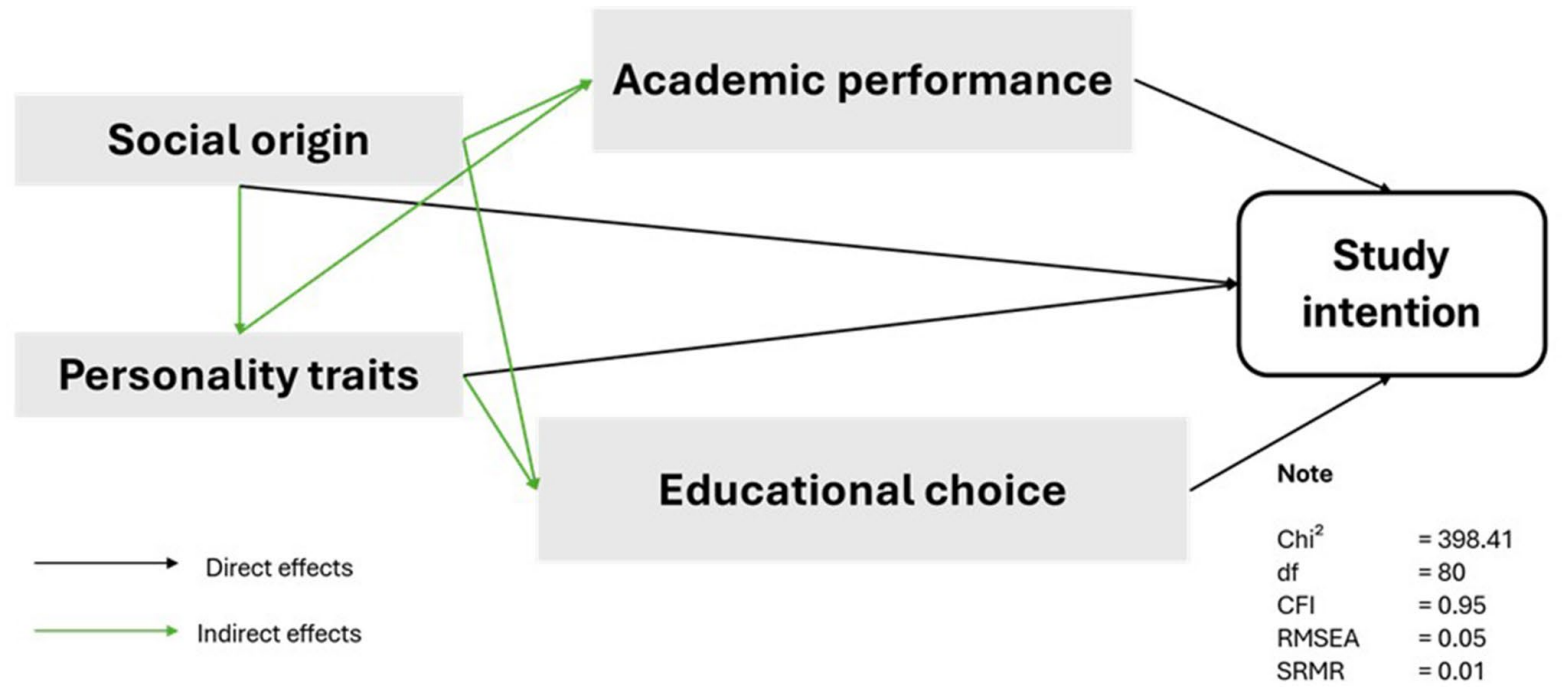
Stylised illustration of path model for SEM. See Supplementary Figure A1 for an illustration of all specified paths, covariances and constraints of the estimated model.

Figure 1 visualises the assumed effects, based on the literature reviewed in this study so far. We expect that the social origin has direct effects on the study intention, as well as mediated effects through academic performance and the variables that measure the educational decision making (secondary effects). Further, we expect the different personality traits have direct effects on the study intention as well as indirect effects, which are mediated via the variables that measure the primary and secondary effects of social origin. Additionally, we consider covariance (1) between the different personality traits, and (2) between the variables that measure the primary and secondary effects of social origin. The control variables (not shown in Figure 1) are considered to have direct effects on the study intention as well as indirect effects mediated through the different personality traits and the average grade.^5^ As noted in Figure 1, our model has a good fit (Kline, 2011; Shi and Maydeu-Olivares, 2020; West et al., 2012; Ximénez et al., 2022).

After the SEM we test for moderation. Therefore, we employ logistic regressions and include interaction terms between the different personality traits and the social origin of the respondents. This allows us to examine, whether group differences of the effects of personality traits on the study intention differ significantly between the three groups. We report average marginal effects as well as predictive margins (Mood, 2010), which allows to interpret percent point differences between the groups as well as changes of the predictive probabilities of the study intention. To adjust for the disproportionality of the sample as well as for the clustering, we use design weights in all our estimations.

We present our results in line with our research questions. In a first step, we observe the bivariate relationship between social background and the independent variables. This is especially important for the interpretation of the effects of personality traits as elaborated in the theory section. Second, we report results of the SEM and test, whether the different personality traits have an effect on the variables which measure the primary and secondary effects of social origin and whether there are group differences. In the third step, we analyse potential differences in the direct, indirect and total effects of personality traits on the study intention. Last, we test for moderation between personality traits and social origin on the study intention.

## 4 Results

### 4.1 Do academic performance and perceptions of success, costs and benefits differ by personality traits?

**Figure 2:**
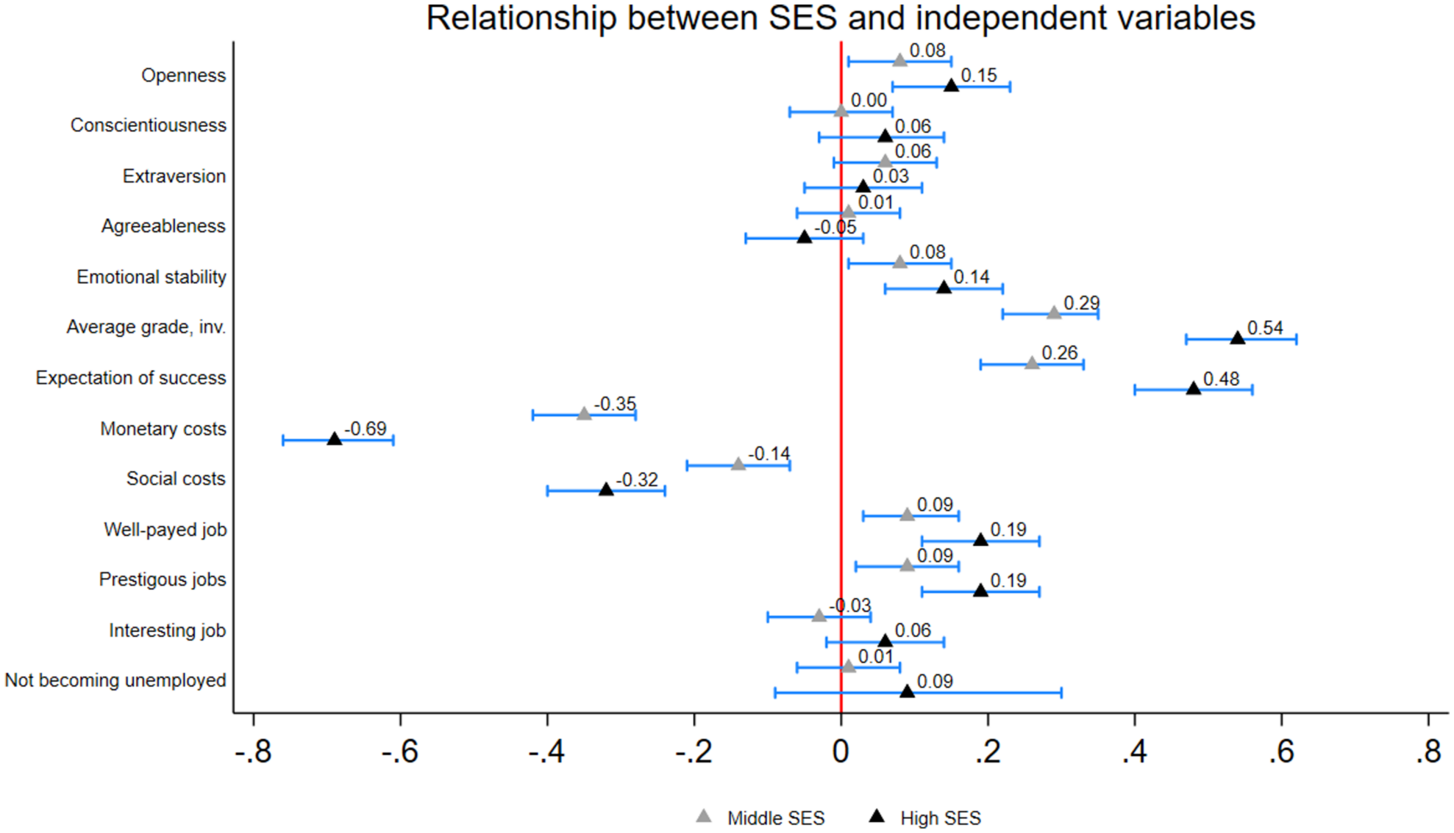
Bivariate relationship between SES and independent variables. Results of bivariate linear regressions. Differences in standard deviations. Reference = low SES. 95% confidence intervals. Weighted results, author’s own calculations.

In a first step, we need to observe the bivariate relationship between SES and our independent variables. As elaborated before, this is important for the theoretical interpretation of the effects of personality traits on the study intention. Figure 2 reports the relationship between SES and the independent variables based on bivariate linear regressions of SES on the different variables. For the personality traits, we observe no significant differences between the low SES group and the other two groups for conscientiousness, extraversion and agreeableness. For openness and emotional stability, we observe slightly higher levels for the middle and high SES groups with a difference of 0.08 and 0.15 standard deviations (SD) for openness and 0.08 and 0.14 SD for emotional stability. Considering the variables that measure the primary and secondary effects of social origin, the group differences are in line with the empirical research and our theory. Individuals with a middle and high SES background have higher grades and a higher expectation of success, their expected costs of studying are lower, and their expected benefits are, in general, higher than for individuals from low SES families.

**Figure 3:**
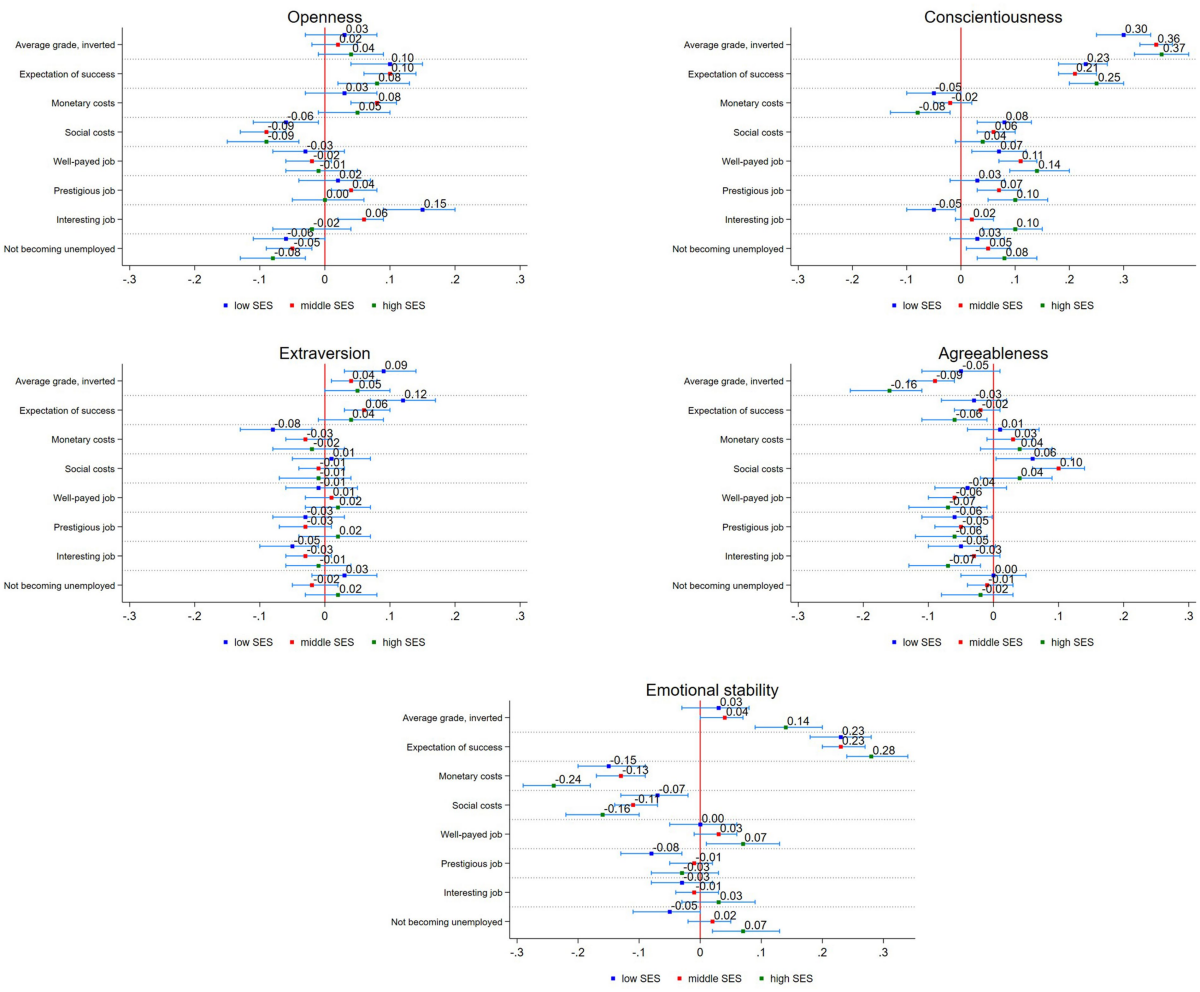
Effects of personality traits on independent variables. Results of SEM. 95% confidence intervals. Author’s own calculations.

In the second step, we test with our SEM whether the personality traits have effects on the variables that measure primary and secondary effects of social origin and whether there are differences between the different social groups (Figure 3). For openness, we observe several significant effects. First, openness has a positive effect on the expectation of success and there are no significant differences by social background (ß = 0.10 for low and middle SES, ß = 0.08 for high SES). Further higher levels of openness seem to increase the perceived monetary costs of studying. However, the effect is only significant for the middle SES group (ß = 0.08). All groups seem to benefit from openness in terms of their perceived social costs, as they are lower with higher openness (ß = −0.06 for low SES; ß = −0.09 for middle and high SES). Further, openness has a positive effect on the expectation of getting a prestigious job through higher education for the middle SES group (ß = 0.04) and negative effects for all groups for the expectation of not becoming unemployed. Interestingly, individuals from low SES families seem to benefit the most from openness for the expectation of getting an interesting job through higher education (ß = 0.15). This effect differs significantly from the high SES group.

As expected from the literature, we observed positive effects from conscientiousness for all groups on the average grades and the expectation of success. For the average grade, the middle (ß = 0.36) and high (ß = 0.37) group benefit slightly more than individuals with a low SES background (ß = 0.30), while there are almost no differences for the expectations of success (ß ranging between 0.21 and 0.25). Further, we observe lower expected monetary costs for the low (ß = −0.05) and high (ß = −0.08) group and higher expected social costs for the low (ß = 0.08) and middle (ß = 0.06) group. Conscientiousness has a positive effect on the expectation of getting a well-payed job for all groups while the perception of getting a prestigious job only significantly differs for the middle (ß = 0.07) and high (ß = 0.10) SES groups. The same accounts for the expectation of not becoming unemployed after higher education. As for openness, we observe significant differences between the low and high SES group for the expectation of getting an interesting job. While there is a negative effect of conscientiousness for the low SES group (ß = −0.05), it is positive for the high SES group (ß = 0.10).

For extraversion, we also observe positive effects of higher levels for the average grade for all groups, however smaller than for conscientiousness. Further, extraversion has a positive effect for the low (ß = 0.12) and middle (ß = 0.06) group on the expectation of success. Interestingly, higher levels of extraversion significantly lower the expected monetary costs of studying for the low SES group (ß = −0.08). Other than that, extraversion has no significant effects on the perceptions of social costs and expected benefits of studying.

Different from the three reported personality traits, higher levels of agreeableness are negatively related with the average grades and the expectation of success for all groups. This accounts especially for the middle (ß = −0.09) and high SES (ß = −0.16) group for the average grade and for expectation of success of the high SES group (ß = −0.06). Apart from that, agreeableness increases the perceived social costs of studying for the low (ß = 0.08) and middle (ß = 0.10) group. Additionally, higher levels of agreeableness are similarly negatively associated with the expected benefits of studying in all three groups.

Lastly for emotional stability, we observe that the high SES group seems to benefit significantly more from higher levels for their average grade (ß = 0.14). Further, emotional stability has a positive effect on the expectation of success for all groups, while the effect is larger for the high SES group (ß = 0.28) than for the other groups (ß = 0.23). Higher levels of emotional stability lower both, the expected monetary and social costs of studying, for all groups. Again, the effects are larger for the high SES group (ß = −0.24 for monetary costs, ß = −0.16 for social costs). Observing the expected benefits from studying, individuals from a high SES background benefit slightly more from higher emotional stability for the expectation of getting a well-payed job (ß = 0.07) than the other groups and significantly more for the perception of not becoming unemployed (ß = 0.07) than the low SES group (ß = −0.05).

### 4.2 Are personality traits more important for children from less advantaged social origin for their study intention?

In the previous section we could observe that personality traits have significant effects on individuals’ academic performance, as well as their perceptions of success, costs and benefits of entering higher education. Aside from that, we could also observe group differences in the effect of the different traits. This leads to assume, that we can observe different indirect effects of personality traits on the study intention, which are mediated by the variables that measure the primary and secondary effects of social origin. This, in turn, leads to the assumption that there are differences in the total effects of the traits on the study intention for each group.

To test this assumption, Table 1 reveals the total, direct and indirect effects of each personality trait on the study intention of our SEM.^6^ The rows for “percentage of total effects mediated by indirect effects” serve as an indicator of the share of indirect effects on the total effects for each personality trait. Overall, we observe that individuals from a low SES background seem to benefit the most from higher levels of openness for their study intention (ß = 0.13), with a slightly weaker effect for middle SES (ß = 0.07) and no effect for the high SES group. The results indicate for the low SES group, that more than half of this total effect is mediated by indirect effects, with the indirect effects of the expectations of success and summarised indirect effects of expected benefits holding the largest share. The share of the expected benefits might largely be explained by the indirect effect of the perception of an interesting job (Supplementary Table T3). The reason for this might be the relatively large positive effect of openness on the expectation of an interesting job for the low SES group (Figure 3).

**Table 1:** Total, direct and indirect effects of personality traits on study intention.

	Low SES	Middle SES	High SES
Openness			
Total effect	0.13***	0.07*	0.00
Direct effect	0.06+	0.02	−0.04
Indirect effects			
Average grade	0.01	0.01	0.01
Expectation of success	0.04***	0.03***	0.03**
Expected costs	0.01	0.01	0.01
Expected benefits	0.02*	0.01	−0.01
Percentage of total effects mediated by indirect effects			
Average grade	7.69%	14.29%	NA
Expectation of success	30.77%	42.86%	NA
Expected costs	7.69%	14.29%	NA
Expected benefits	15.38%	14.29%	NA
Conscientiousness			
Total effect	0.10**	0.13***	0.19***
Direct effect	−0.07+	−0.06*	−0.08+
Indirect effect			
Average grade	0.09***	0.11***	0.12***
Expectation of success	0.08***	0.07***	0.08***
Expected costs	−0.01+	0.01+	0.00
Expected benefits	0.01	0.02***	0.06***
Percentage of total effects mediated by indirect effects			
Average grade	90.00%	84.61%	63.16%
Expectation of success	80.00%	53.85%	42.11%
Expected costs	−10.00%	7.69%	0.00%
Expected benefits	10.00%	15.38%	31.58%
Extraversion			
Total effect	0.06	0.03	0.04
Direct effect	−0.02	−0.02	0.00
Indirect effect			
Average grade	0.03**	0.01*	0.02+
Expectation of success	0.04***	0.02***	0.01
Expected costs	0.00	0.00	0.00
Expected benefits	0.00	0.01	0.00
Percentage of total effects mediated by indirect effects			
Average grade	50.00%	33.33%	50.00%
Expectation of success	66.67%	66.67%	25.00%
Expected costs	0.00%	0.00%	0.00%
Expected benefits	0.00%	33.33%	0.00%
Agreeableness			
Total effect	−0.14***	−0.08**	−0.16***
Direct effect	−0.09**	−0.01	−0.05
Indirect effect			
Average grade	−0.01+	−0.03***	−0.05***
Expectation of success	−0.01	−0.01	−0.02*
Expected costs	−0.01+	−0.01***	−0.00
Expected benefits	−0.02*	−0.02**	−0.04***
Percentage of total effects mediated by indirect effects			
Average grade	7.14%	37.50%	31.25%
Expectation of success	7.14%	12.50%	12.50%
Expected costs	7.14%	12.50%	0.00%
Expected benefits	14.29%	25.00%	25.00%
Emotional stability			
Total effect	0.05	0.02	0.04
Direct effect	−0.04	−0.08**	−0.15**
Indirect effect			
Average grade	0.01	0.01	0.05***
Expectation of success	0.08***	0.07***	0.09***
Expected costs	0.02*	0.02***	0.02
Expected benefits	−0.01	0.00	0.02+
Percentage of total effects mediated by indirect effects			
Average grade	20.00%	50.00%	125.00%
Expectation of success	160.00%	350.00%	225.00%
Expected costs	40.00%	100.00%	50.00%
Expected benefits	−20.00%	0.00%	50.00%
*N*	1,468	2,997	1,412

DZHW Panel Study of School Leavers 2018, author’s own calculations from SEM. Effects for expected costs and benefits are summarised (see Supplementary Tables T2–T5). + *p* < 0.10, * *p* < 0.05, ** *p* < 0.01, *** *p* < 0.001. Controlled for gender and migration background.

For conscientiousness, the group of individuals with a high SES background seems to benefit the most from higher levels of this trait (ß = 0.19) for their study intention, compared to the groups from low (ß = 0.10) and middle SES (ß = 0.13). We can observe for all three groups, that the share of the indirect effects on the total effects exceeds that of the direct effect of conscientiousness. The comparatively large indirect effects can mostly be explained by the indirect effects of the average grade and the expectation of success, which can be explained by the positive effects of conscientiousness on these variables for all groups (Figure 3).

Higher levels of agreeableness have negative total effects on the study intention for all three groups. Individuals from high SES backgrounds have the largest negative total effect (ß = −0.16), followed by the groups of low (ß = −0.14) and middle SES (ß = −0.08). For the high SES group, this effect is mainly driven by the negative indirect effects of the average grade (ß = −0.05) and expected benefits (ß = −0.04). The negative effects of the expected benefits are mainly driven by the indirect effects of getting a well-payed job and an interesting job (Supplementary Table T4). However, the total effect for the low SES group is mainly driven by the negative direct effect (ß = −0.09), while the lower negative total effect for the middle SES group, can mainly be explained due to the low direct effect (ß = −0.01).

Considering extraversion, positive albeit not significant, total effects can be observed for all three groups. Although there are slight positive indirect effects of the average grade and expectation of success, almost no direct effects can be observed for any of the group, which explains the absence of significant total effects. Lastly, no significant total effect on the study intention can be observed for emotional stability. Although we can observe positive indirect effects for all groups, which are driven by positive indirect effects of the expected success and expected costs, the total effects are reduced by the negative direct effects of emotional stability in all groups.

**Figure 4:**
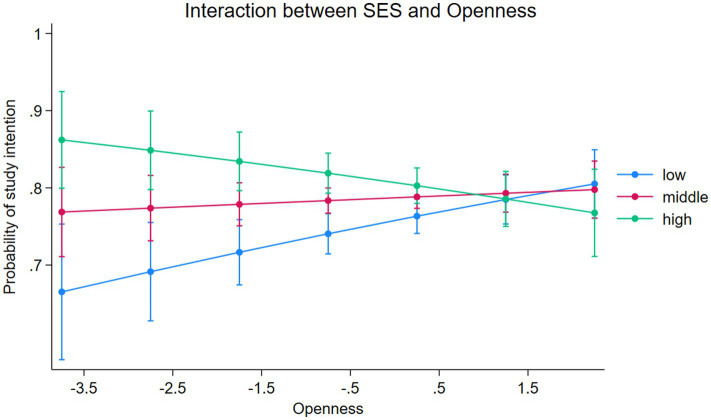
Probability of study intention (predictive margins) for the interaction between SES and openness. Results from logistic regression (see Supplementary Table T6). 95% confidence intervals. Weighted results, author’s own calculations.

The SEM revealed differences in the total effects of personality traits on the study intention between the different groups. We found the most pronounced differences in the total effects of openness and conscientiousness. To test whether these differences are significant, we employ a logistic regression model to test for moderation (see Supplementary Table T6). With this method, we can additionally control for interaction terms between social origin and each personality trait. The results reveal only one significant interaction. It shows that the effect of openness is about 4 % points stronger for individuals with a low SES background than for individuals from high SES families. To better understand the differences of the effect of openness, Figure 4 illustrates the predictive margins of the study intention for different levels of openness for each group. We can see the strongest positive effect for individuals with a low SES background and a negative effect for individuals from high SES families. While individuals from low SES families with a relatively low openness have a predicted probability of having a study intention of about 66.5%, it increases up to about 80.5% with a relatively high openness. This means that higher levels of openness can increase the probability of having a study intention up to 14 percent points for children from low SES families.

## 5 Discussion

This research aimed to explore how high school graduates from less advantaged social origins can overcome social inequalities in higher education enrolment. The reason for investigating is that sociological research to date has mainly focused on a deficit perspective, asking why there are social inequalities at the transition into higher education. However, how some high school graduates with less advantaged social origins are still able to transition into higher education against the odds is an underrepresented topic in research. Derived from a resource perspective we complement the sociological perspective by including the Big Five personality traits as resources that individuals can rely on for their educational decision-making. According to resource theories we expected three different mechanisms: *resource substitution*, structural amplification, and resource multiplication or Matthew effect. Using representative German data from the DZHW 2018 Panel Study of School Leavers, we used structural equation modelling (SEM) to model the effects of personality traits on variables associated to the primary and secondary effects of social origin as well as direct, indirect and total effects of personality traits on the intention to enrol into higher education. While the influence of personality on school performance (primary effect) is well examined in educational economics and psychology (Vedel, 2014), the influence on educational decision-making (secondary effects) is not. This is where our article aimed to add value.

First, the results have shown that personality traits have significant effects on the academic performance as well as the perceived costs, benefits and the expected success in higher education. However, how these indicators are affected, differs largely for the different traits. Second, the total effects of personality traits on the study intention reveal that different mechanisms are involved. The most pronounced effects could be found for openness. The moderation analysis revealed that individuals from low origin families benefit significantly more from higher levels of openness for their study intention than individuals from a high social origin. These results are in line with past research (Peter and Storck, 2015; Shanahan et al., 2014). The mediation analysis has shown that this can partly be explained by larger indirect effects via expected success and benefits. For the expected benefits, the expectation of getting an interesting job with a higher education degree seems to be particularly important for low origin graduates, as openness shows a significantly larger effect for this group. Despite individuals from a social disadvantaged background benefitting more from higher openness, the bivariate analysis revealed that this group has lower levels of openness in general, compared to the groups of more advantaged backgrounds. According to the theoretical assumptions, the positive effect of openness can be described as *resource substitution* but ends up as a mechanism of structural amplification (Ross and Mirowsky, 2011). Therefore, openness can serve as a mechanism of mobility for some individuals from lower social origins but in sum functions as a mechanism of the reproduction of social stratification. This means that, although individuals with a disadvantaged background can benefit more from higher levels of openness, they can make lest often used of it, due to having lower endowments of this resource in general. For conscientiousness, the results of the total effects revealed that individuals from higher social strata might benefit more from this resource. According to the Matthew effect (Merton, 1968) conscientiousness might help this groups to benefit even more for entering higher education, since they have already more from the other important resources (for example better academic performance). However, moderation analysis revealed that this differences of the effect of conscientiousness on the study intention are not statistically significant. For agreeableness, the results revealed similar negative effects on the study intention for all groups, while no relevant total effects of extraversion and emotional stability could be found.

Based on these results, we argue that children with less advantaged social origins can overcome social inequalities in higher education enrolment due to becoming more open. However, since they are on average less open, the question remains as to whether this personality trait could be strengthened, to improve the chances for this group of enrolling in higher education. There is some evidence pointing to the variability of these personality traits and giving hints for potential interventions (Stieger et al., 2020; Zimmermann and Neyer, 2013). Overall, our results show the importance of taking non-cognitive resources like personality traits into account when researching social inequalities in educational decision-making.

Despite these substantial results, our research also has some limitations. First, all our variables were measured at the same time. This makes it difficult to speak of causal relationships between the variables. Second, due to data availability, for this research we focused on the effects on the intention to enrol in higher education. Although there is a high correlation between the intention and the actual transition into higher education in Germany, results of the effects of personality might still differ. This limitation can be solved when more waves of the survey become available so that the effect on the actual transition into higher education can be measured. Further, we used the International Socioeconomic Index of Occupation (ISEI) as the measurement for the social origin of high school graduates, which was measured in the second wave of the survey. The data of this wave was collected about 6 months after the first wave when the study intention was measured. Thus, uncertainty remains as to how far the results are affected due to panel mortality.

To address these limitations, further research might use panel data over a longer timeframe to identify the causal effects of personality on the actual transition into higher education for high school graduates with different social origins. Further, this data would need to measure personality before the respective variables associated to primary and secondary effects of social origin. This would allow for a causal analysis of personality traits on these variables. Last, the analysis of the effect of personality for different groups of social origin on different outcomes, such as the subject of study or the study’s success, might also be interesting starting points for further research.

## Data Availability

The data analysed in this study is subject to the following licenses/restrictions: the data of the first wave of the 2018 cohort used in the empirical analyses are available from the Research Data Centre for Higher Education Research and Science Studies (fdz.DZHW). Data of the second wave will become available in the future. For information on terms and conditions, please refer to https://www.fdz.dzhw.eu/en. Requests to access these datasets should be directed to https://www.fdz.dzhw.eu/en.

## Footnotes

^1^The share of respondents with a study intention closely aligns with official statistics of actual higher education enrolments for this cohort (Authoring Group Educational Reporting, 2024, Tab. F2-3web).

^2^In Germany, the Abitur is the qualification awarded upon completion of upper secondary education and serves as the main requirement for university entrance. Students receive a final grade that reflects the weighted average of all subject grades over the last 2 years of schooling. This final grade is highly relevant for higher education access, as many university programmes in Germany have restricted admissions (Numerus clausus). In our study, we used self-reported average school grades collected 6 months prior to graduation. Respondents were asked to report their current grade point average at the time of the survey, which coincided with the measurement of their intention to study. This approach ensures that the academic performance indicator is temporally aligned with the decision-making process and captures the level of achievement that typically determines eligibility for university admission.

^3^Our selection of indicators is based on the study by Quast et al. (2023), who demonstrate that all four components significantly influence the decision to enter higher education. Importantly, their decomposition analyses show that social differences exist for all four of these indicators.

^4^See Supplementary Table T1 for an overview of all variables.

^5^We are aware that our model suggests causal assumptions, which cannot be tested with our data. For this, longitudinal data over a long timeframe would be needed. However, as already elaborated in this paper, it is based on strong theoretical assumptions and empirical evidence and should therefore deliver a good insight into the complex relationship between the model variables.

^6^For illustration purposes, the effects of the variables for expected costs and benefits are summarised. For a presentation of the detailed effects, see Supplementary Tables T3–T5.
