# Supplementary material for: Social origin and the intention to enrol in higher education: personality traits as a mechanism of reproduction or mobility?
Source: Front Sociol. 2025 Aug 22;10:1652429. doi: 10.3389/fsoc.2025.1652429 (PMC12412136; doi:10.3389/fsoc.2025.1652429)
Supplement: Supplementary file 1 [file Supplementary_file_1.docx]

# **Supplementary material**

**Tab T1. Overview of independent variables**

| **Variable** | **Measurement instrument** | **Level of measurement** | **%** |
| --- | --- | --- | --- |
| **Big Five personality traits** | Big Five Inventory-SOEP (BFI-S) | metric 1-7 (z-standardized) |  |
| openness | (Schupp & Gerlitz 2008) |  |  |
| conscientiousness |  |  |  |
| extraversion |  |  |  |
| agreeableness |  |  |  |
| emotional stability |  |  |  |
| **Social background** | HISEI score: highest ISEI score of | categorical (low, middle, |  |
| low | both parents (Ganzeboom 2010) | high) | 26.43% |
| middle |  |  | 50.72% |
| high |  |  | 22.85% |
| **average grade, inv.** | Please indicate your current grade point average. | metric (z-standardized) |  |
| **expected success** | How high do you rate your chances of successfully completing a university degree? | metric 1-5 (z-standardized) |  |
| **monetary costs (α=0.66) (mean index)** | During university studies, various expenses must be paid, e.g., travel costs, books, or fees. How difficult would it be for you and your family to cover these costs if you were to start studying? | metric 1-5 (z-standardized) |  |
|  | Similarly, during a degree program, you can only earn a limited amount of money. If you were to start a degree program, how high would be your income loss? | metric 1-5 (z-standardized) |  |
|  | Do the costs of studying influence your decision for or against studying? | metric 1-5 (z-standardized) |  |
| **social costs (α=0.80) (mean index)** | How important are the following aspects to you when choosing your future place of work or study? |  |  |
|  | Parents, relatives, or friends live at the place of training/study | metric 1-5 (z-standardized) |  |
|  | Proximity to hometown | metric 1-5 (z-standardized) |  |
| **well-paying job (sum index)** | How good do you think your chances would be of getting a well-paid job later on, |  |  |
|  | if you completed vocational training? | metric 1-5 (z-standardized) |  |
|  | if you completed a degree? | metric 1-5 (z-standardized) |  |
| **prestigious job (sum index)** | How good do you think your chances would be of getting a prestigious job later on, |  |  |
|  | if you completed vocational training? | metric 1-5 (z-standardized) |  |
|  | if you completed a degree? | metric 1-5 (z-standardized) |  |
| **interesting job (sum index)** | How good do you think your chances would be of getting an interesting job later on, |  |  |
|  | if you completed vocational training? | metric 1-5 (z-standardized) |  |
|  | if you completed a degree? | metric 1-5 (z-standardized) |  |
| **not becoming unemployed (sum index)** | How good do you think your chances would be of not becoming unemployed later on, | metric 1-5 (z-standardized) |  |
|  | if you completed vocational training? |  |  |
|  | if you completed a degree? | metric 1-5 (z-standardized) |  |
| **gender** | Which gender do you have? | categorical |  |
| male |  |  | 34.96% |
| female |  |  | 65.04% |
| **migration background** | Birthplace of respondent and parents | categorical |  |
| no |  |  | 84.39% |
| yes |  |  | 15.61% |

Source: DZHW Panel Study of School Leavers 2018 N=5,877. Metric variables are z-standardised and thus, have a mean of 0 and a standard deviation of 1. Weighted results. Author’s own calculations.

**Tab. T2 Direct effects on study intention**

|  | low SES | middle SES | high SES |
| --- | --- | --- | --- |
| openness | 0.06+ | 0.02 | -0.04 |
| conscientiousness | -0.07+ | -0.06* | -0.08+ |
| extraversion | -0.02 | -0.02 | 0.00 |
| agreeableness | -0.09** | -0.01 | -0.05 |
| emotional stability | -0.04 | -0.08** | -0.15** |
| average grade, inv. | 0.29*** | 0.31*** | 0.33*** |
| expectation of success | 0.37*** | 0.31*** | 0.33*** |
| monetary costs | -0.04 | -0.08** | -0.02 |
| social costs | -0.12*** | -0.11*** | -0.09* |
| well-payed job | 0.18*** | 0.13*** | 0.16*** |
| prestigious job | 0.05 | 0.02 | 0.09* |
| interesting job | 0.17*** | 0.21*** | 0.24*** |
| not becoming unemployed | 0.01 | 0.04 | 0.09+ |
| gender | -0.13*** | -0.04 | -0.12* |
| migration background | 0.15*** | 0.13*** | 0.13** |
| **N** | 1468 | 2997 | 1412 |

Source: DZHW Panel Study of School Leavers 2018, authors own calculations from SEM. + p<0.10, * p<0.05, ** p<0.01, *** p<0.001. Controlled for gender and migration background.

**Tab. T3 Indirect effects of personality traits on study intention through mediation for low SES**

|  | average grade, inv. | expectation of  success | monetary costs | social costs | well-payed job | prestigious job | interesting job | not becoming unemployed |
| --- | --- | --- | --- | --- | --- | --- | --- | --- |
| openness | 0.01 | 0.04*** | 0.00 | 0.01+ | -0.01 | 0.00 | 0.03*** | 0.00 |
| conscientiousness | 0.09*** | 0.08*** | 0.00 | -0.01* | 0.01* | 0.00 | -0.01* | 0.00 |
| extraversion | 0.03** | 0.04*** | 0.00 | 0.00 | 0.00 | 0.00 | 0.00 | 0.00 |
| agreeableness | -0.01+ | -0.01 | 0.00 | -0.01+ | -0.01 | 0.00 | -0.01+ | 0.00 |
| emotional stability | 0.01 | 0.08*** | 0.01 | 0.01* | 0.00 | 0.00 | -0.01 | 0.00 |

Source: DZHW Panel Study of School Leavers 2018, authors own calculations from SEM. + p<0.10, * p<0.05, ** p<0.01, *** p<0.001. N=1468.

Controlled for gender and migration background.

**Tab. T4 Indirect effects of personality traits on study intention through mediation for middle SES**

|  | average grade, inv. | expectation of  success | monetary costs | social costs | well-payed job | prestigious job | interesting job | not becoming unemployed |
| --- | --- | --- | --- | --- | --- | --- | --- | --- |
| openness | 0.01 | 0.03*** | -0.01* | 0.01*** | 0.00 | 0.00 | 0.01** | 0.00 |
| conscientiousness | 0.11*** | 0.07*** | 0.00 | -0.01** | 0.01*** | 0.00 | 0.01 | 0.00 |
| extraversion | 0.01* | 0.02*** | 0.00 | 0.00 | 0.00 | 0.00 | 0.01 | 0.00 |
| agreeableness | -0.03*** | -0.01 | 0.00 | -0.01*** | -0.01** | 0.00 | -0.01+ | 0.00 |
| emotional stability | 0.01 | 0.07*** | 0.01** | 0.01*** | 0.00 | 0.00 | 0.00 | 0.00 |

Source: DZHW Panel Study of School Leavers 2018, authors own calculations from SEM. + p<0.10, * p<0.05, ** p<0.01, *** p<0.001. N=2997.

Controlled for gender and migration background.

**Tab. T5 Indirect effects of personality traits on study intention through mediation for high SES**

|  | average grade, inv. | expectation of  success | monetary costs | social costs | well-payed job | prestigious job | interesting job | not becoming unemployed |
| --- | --- | --- | --- | --- | --- | --- | --- | --- |
| openness | 0.01 | 0.03** | 0.00 | 0.01+ | 0.00 | 0.00 | -0.01 | -0.01 |
| conscientiousness | 0.12*** | 0.08*** | 0.00 | 0.00 | 0.02** | 0.01+ | 0.02** | 0.01 |
| extraversion | 0.02+ | 0.01 | 0.00 | 0.00 | 0.00 | 0.00 | 0.00 | 0.00 |
| agreeableness | -0.05*** | -0.02* | 0.00 | 0.00 | -0.01+ | -0.01 | -0.02* | 0.00 |
| emotional stability | 0.05*** | 0.09*** | 0.01 | 0.01+ | 0.01+ | 0.00 | 0.01 | 0.01 |

Source: DZHW Panel Study of School Leavers 2018, authors own calculations from SEM. + p<0.10, * p<0.05, ** p<0.01, *** p<0.001. N=1412.

Controlled for gender and migration background.

|  | **AME** |
| --- | --- |
| **SES (ref. high SES)** |  |
| low | -0.05*** |
| middle | -0.02 |
| **Personality traits** |  |
| openness | 0.01 |
| conscientiousness | -0.02** |
| extraversion | -0.01 |
| agreeableness | -0.02** |
| emotional stability | -0.02*** |
| **Personality x social origin (ref. high SES)** |  |
| openness x low SES | 0.04* |
| openness x middle SES | 0.02 |
| conscientiousness x low SES | -0.01 |
| conscientiousness x middle SES | -0.01 |
| extraversion x low SES | 0.00 |
| extraversion x middle SES | 0.00 |
| agreeableness x low SES | -0.01 |
| agreeableness x middle SES | 0.02 |
| emotional stability x low SES | 0.01 |
| emotional stability x middle SES | 0.00 |
| **Primary effect of social origin** |  |
| average grade, inv. | 0.09*** |
| **Secondary effect of social origin** |  |
| expected success | 0.10*** |
| monetary costs | -0.01* |
| social costs | -0.03*** |
| well-payed job | 0.04*** |
| prestigious jobs | 0.02*** |
| interesting job | 0.06*** |
| not becoming unemployed | 0.01+ |
| **Controls** |  |
| gender (ref. male) | -0.01 |
| migration background (ref. no) | 0.08*** |
| **Pseudo-R²** | 0.29 |
| **N** | 5877 |

**Tab. T6 Logistic regression on study intention with interaction terms**

Source: DZHW Panel Study of School Leavers 2018, authors own calculations. AME= Average Marginal Effect. + p<0.10, * p<0.05, ** p<0.01, *** p<0.001.


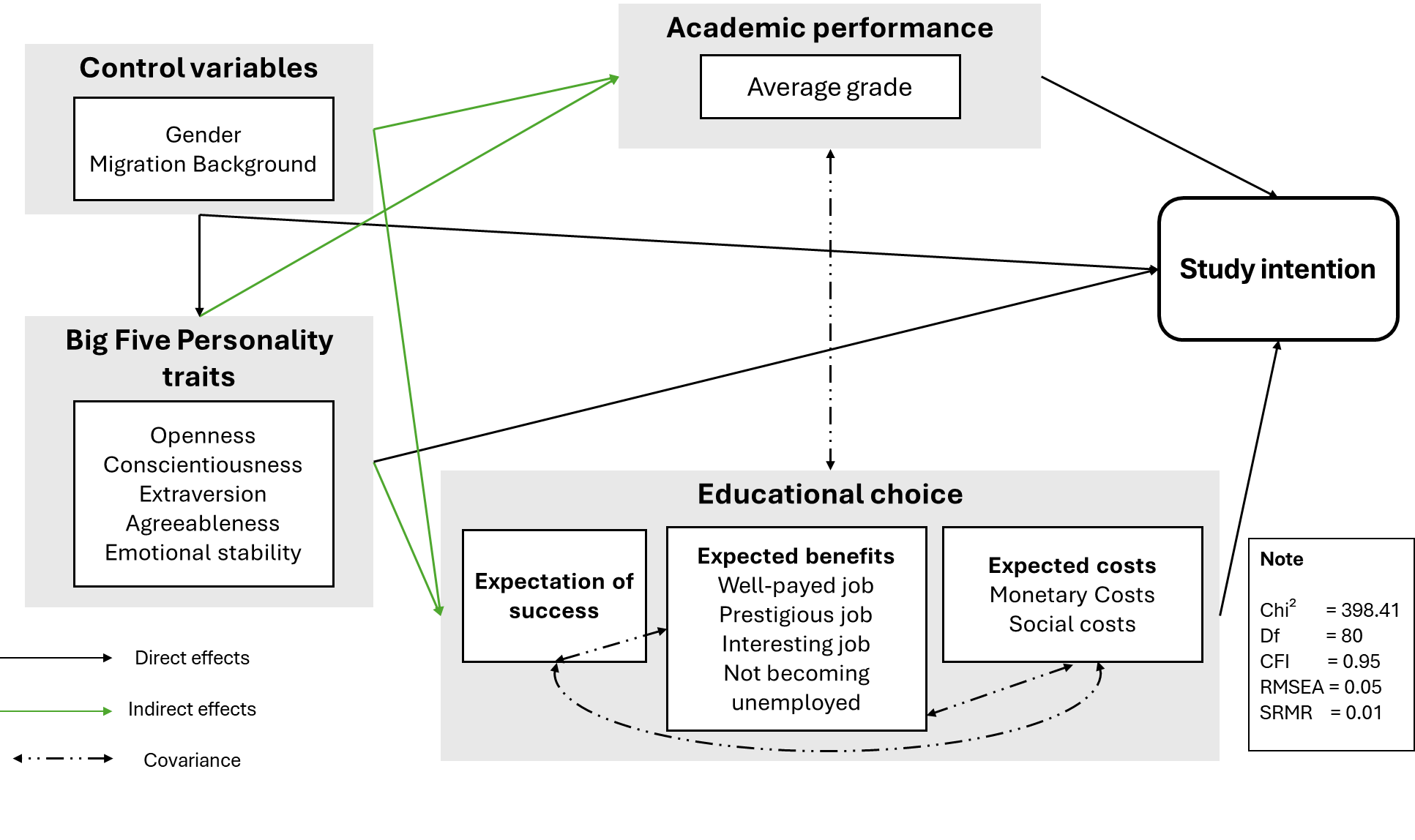


**Figure A1** Path model. Overview over all specified paths for each group in the structural equation model. Note: Additional covariances are considered between (1) each personality trait, (2) between each benefit variable and (3) between each cost variable.
